# Prevalence of *Listeria monocytogenes* infection in women with spontaneous abortion, normal delivery, fertile and infertile

**DOI:** 10.1186/s12884-022-05330-6

**Published:** 2022-12-28

**Authors:** Amjad Ahmadi, Rashid Ramazanzadeh, Safoura Derakhshan, Mazaher Khodabandehloo, Fariba Farhadifar, Daem Roshani, Atefeh Mousavi, Manouchehr Ahmadi Hedayati, Mohammad Taheri

**Affiliations:** 1 Social Determinants of Health Research Center, Research Institute for Health Development, Kurdistan University of Medical Sciences, Sanandaj, Iran; 2 Department of Microbiology, School of Medicine, Hamadan University of Medical Sciences, Hamadan, Iran; 3 Department of Microbiology, Faculty of Medicine, Ardabil University of Medical Sciences, Ardabil, Iran; 4 Liver and Digestive Research Center, Research Institute for Health Development Kurdistan University of Medical Sciences, Sanandaj, 66177-13446 Iran; 5 Department of Microbiology, Faculty of Medicine, Kurdistan University of Medical Sciences, Sanandaj, Iran

**Keywords:** *Listeria monocytogenes*, Spontaneous abortion, Infertility

## Abstract

**Background:**

*Listeria monocytogenes* with a vast range of natural reservoirs is more known for being a food-borne pathogen. Human infections have shown an impact on pregnancy outcomes, so, this study surveyed the frequency of *L. monocytogenes* infection involving different groups of women.

**Methods:**

This study enrolled a total sample consisting of 109 women with spontaneous abortion, 109 women with normal delivery, 100 fertile women, and 99 infertile women aged 19–40 years and willing to participate in the study. The research tool in this study was a questionnaire and Polymerase chain reaction (PCR) test.

**Results:**

According to the results, the frequency of *L. monocytogenes* infection was 4/109 (3.66%) observed among women with spontaneous abortion, 2/109 (1.83%) among women with normal delivery, 3/100 (3%) among fertile women, and 0/99 (0%) among infertile women.

**Conclusion:**

There was no significant relationship between *Listeria monocytogenes* infection and pregnancy outcomes of spontaneous abortion and infertility.

## Introduction

*Listeria monocytogenes* is known as a non-spore forming, non-branching, regular, short rod, gram-positive, and facultative anaerobic bacterium isolated from soil, animal food, water, feces, animals, and humans. Since it can grow at a temperature of 4°C, refrigerated food should be taken into consideration as a potential source of infections [1–3]. Since 1980, many cases of *L. monocytogenes* infection have been reported as a series of epidemic or sporadic infections due to the consumption of contaminated food [4]. Since the bacterium is ubiquitous, efforts to prevent contamination of sources should be stepped up by further controlling on chains of producing and distributing food [5, 6]. Previous studies in several countries have also reported a high potential risk of bacterial-related contamination in dairy and meat products [5, 6].

The relevant studies show bacterial infection can involve pregnant women, infants, and immunocompromised patients with a variety of clinical complications including meningitis, septicemia, miscarriage, stillbirth, or meningoencephalitis [7–9]. In addition, in non-pregnant women, *L. monocytogenes* causes primary meningitis, encephalitis, and septicemia [10]. Studies show elderly and immunocompromised people involving transplant recipients, lymphoma, and acute immunodeficiency syndrome (AIDS) are more prone to *L. monocytogenes* infections [11]. *L. monocytogenes* infections tend to invade the central nervous system leading to acute diseases usually with a high mortality rate and lasting neurological sequelae [10, 11]. According to previous reports, pregnancy situation increases the risk of listeriosis following passing bacterium through the placenta causing a bacteremia that without treatments can lead to inflammation of the placenta or amniotic sac, fetal infection, and consequently miscarriage, stillbirth, or premature birth [10, 12–14]. In the last two decades, the use of vaginal samples to diagnose sexually transmitted infections (STIs) has increased, however, it has never been used to diagnose *L. monocytogenes* in abortion cases [15]. The relevant studies show that high rates of abortions have also been reported via home calls for patients with clinical infections [15]. Despite considering *L. monocytogenes* as a part of the faecal microbiota in most mammals, up to 5% of healthy animals should be taken into consideration as the asymptomatic carriers [16]. It seems that the study on human (as the permanent reservoir of *L. monocytogenes*) microbiota samples including intestines, vagina, milk, and urine have not taken into consideration as well as human-to-human transmission routs [16–18]. Given the importance of *L. monocytogenes* infection connected to pregnant women and its consequences in pregnancy, this study, based on the type of samples, used the vaginal swabs to detect *L. monocytogenes* infections. The possible results would allow obstetricians and gynecologists to get better perspective of appropriate diagnostic prenatal tests.

## Materials and Methods

### Sample selection

Samples were collected from women referred to the Obstetrics and Gynecology Clinic at Besat Hospital in Sanandaj, Iran. Information about individuals was collected through a checklist in patient records. Data such as age, location, education, smoking, unpasteurized dairy consumption, history of genital infection, and local dairy consumption history were extracted. Informed consent was obtained from all participants in this study. According to the reported prevalence of *Listeria monocytogenes* infections in Sattari et al [19], the sample size was 417 (with 95% confidence and 5% accuracy). This study was performed on the vaginal swab samples of 109 women with spontaneous abortion in a range of gestational time between 10-20 weeks, 109 women with normal delivery with a gestational period from 37 and up, and 100 fertile women and 99 infertile women aged 19–40 years who were appealed to participate in this study.

The Sampling was done in the subject groups including four women groups with abortion, natural childbirth, fertile and infertile. Vaginal swab samples were collected from women with abortion symptoms right before an abortion due to discharge and washing the vagina, perineum and cervix with using betadine and in some cases due to the use of antibiotics. Samples with normal delivery were obtained before the rupture of the fetal water sac and at the time of the onset of labor pain in the delivery room. In addition, the samples of two fertile and infertile groups were obtained at the time of their visit to the women's clinic. The infertility of women was examined by using spermogram test and a failure in fertility following sexual contact with unprotected men. The research tools in this study were a questionnaire and polymerase chain reaction (PCR) test.

### DNA extraction

Falcon tubes containing the swab samples of individuals were collected in phosphate-buffered saline (CinnaGen, Tehran, Iran) and then stored at -20°C until extraction. They were then centrifuged at 2000 rpm for 15 min. The supernatant was then discarded, and the precipitate was transferred to 1.5 ml microtubes and centrifuged again at 2000 rpm for 15 min. The supernatant was discarded again and the precipitate was used to extract DNA kit (High pure PCR Template Preparation; Roche, Germany). DNA extraction steps were performed according to the Kit instructions. Extracted DNA samples were stored in 1.5 ml microtubes at -20°C until PCR.

### PCR assay

To begin with, *hlyA* gene specific primers were designed by using the primer3 online. The *hlyA* gene primers of *L. monocytogenes* were compared with complete genomes of other strain GenBank such as LM series, S10, S12, BR, BS and etcetera. The primers showed 100 percent query cover with complementary genome regions. The specificity and sensitivity of primers were obtained by using an exact comparison of primers on the BLAST website and using standard strain, respectively. The primer sequences were as follows: Forward: 5′- F: GCTGAAGAGATTGCGAAAGAAG-3′ and Reverse: 5′-CAAAGAAACCTTGGATTTGCGG -3′. The length of the PCR target was 370 bp. The PCR reactions were performed in a total volume of 25 μL containing PCR Master Mix (CinnaGen, Tehran, Iran).

### PCR amplification

The PCR amplification program (Eppendorf, Hamburg, Germany) was as follows: Initial denaturation at 94°C for 5 min, followed by 30 cycles of denaturation at 94°C for 30 sec, annealing at 58°C for 30 sec, extension at 72°C for 45 sec, and final extension at 72°C for 5 min. The PCR products were separated by electrophoresis on 1.5% agarose gel (CinnaGen, Tehran, Iran) stained with ethidium bromide, and visualized under ultraviolet (UV) light. The standard strain of *L. monocytogenes* as positive control was prepared from the Iranian Biological Resource Center (Strain Number: IBRC-M 10671, another collection number: ATCC 13932, LMG 21264, and NCTC 10527). All positive PCR tests with a product size similar to standard strains̓ PCR product size were considered positive, in addition, the master mix without the DNA template was used as the negative control. We used the 0.5 McFarland standard dilutions to obtain the concentration of 10-15 colony forming units (CFU) per mL for bacteria colony counting, DNA extraction and PCR to accredit the sensitivity of PCR (with a detection limit of 150 CFU/mL).

### Statistical analysis

Data were entered into SPSS (ver. 20) and presented as percentage and mean in tables and diagrams. Quantitative values were stated as mean ± standard deviation. The Fisher’s exact test, *t-* test, and Chi-square test were used to compare qualitative variables between the two groups. P<0.05 was considered statistically significant.

## Results

**Fig. 1:**
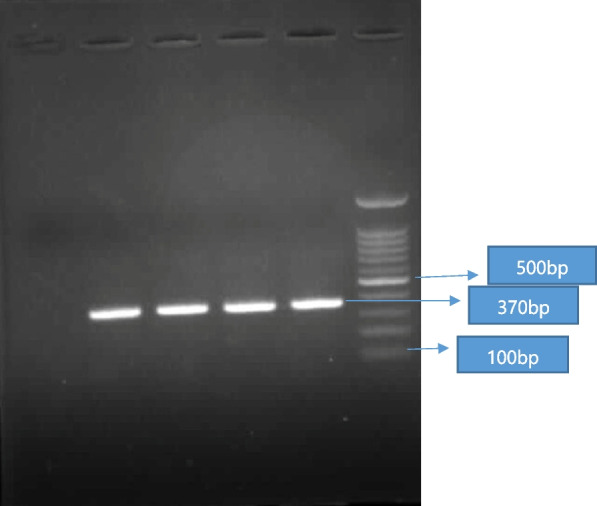
Polymerase chain reaction (PCR) assay for *Listeria monocytogenes* detection. Lane 1: 100-bp DNA ladder (SinaClon, Tehran, Iran); lane 2: PCR-positive control (370 bp); Lane 3-5: positive PCR products; Lanes 6: negative control

The mean age of women with spontaneous abortion was (29.6 ± 5.9) and the mean age of women with normal delivery was (27.8 ± 4.87). Unpasteurized dairy consumption was reported in 2/109 (1.83%) women with spontaneous abortion in 3/109 (2.75%) women with normal delivery in 4/100 (4%) fertile women, and in 1/99 (1.01%) infertile women. The frequency of *L. monocytogenes* infection was 4/109 (3.66%) observed among women with spontaneous abortion, 2/109 (1.83%) among women with normal delivery, 3/100 (3%) among fertile women, and 0/99 (0%) among infertile women. The highest unpasteurized dairy consumption was in women with normal delivery 3/109 (2.75%) and fertile women 3/100 (3%). The highest smoking rate was for infertile women 15/99 (15.15%). The results showed that there was no association between *Listeria monocytogenes* infection and spontaneous abortion and infertility (*p*-value>0.05). Tables 1 and 2 present the complete results of the study. Figure 1 presents the PCR results and band patterns.

**Table 1:** Demographic data of *Listeria monocytogenes* infections in women with spontaneous abortion and normal delivery

Variables		Spontaneous abortion *n*=109	Normal delivery *n*=109	*p*-value
Age		(29.6 ± 5.9)	(27.8 ± 4.87)	0.83
Education	Illiterate	5 (4.58%)	3 (2.75%)	0.22
	School education	85 (77.98%)	77 (70.64%)	
	University education	19 (17.43%)	29 (26.60%)	
Location	City	87 (79.81%)	81 (74.31%)	0.33
	Village	22 (20.18%)	28 (25.68%)	
Smoking		0 (0%)	3 (2.75%)	0.43
Unpasteurized dairy consumption		2 (1.83%)	3 (2.75%)	0.50
History of genital infection		11 (10.09%)	5 (4.58%)	0.115
Local dairy consumption history		8 (7.33%)	9 (8.25%)	0.80
*Listeria monocytogenes*		4 (3.66%)	2 (1.83%)	0.40

**Table 2:** Demographic data of *Listeria monocytogenes* infections in fertile women and infertile women

Variables		Fertility *n*=100	Infertility *n*=99	*p*-value
Age		(32.1 ± 5.1)	(29.1 ± 6.3)	0.00
Education	Illiterate	41 (41%)	47 (47.47%)	0.094
	School education	42 (42%)	46 (46.47%)	
	University education	17 (17%)	7 (7.07%)	
Smoking		4 (4%)	15 (15.15 %)	0.007
Location	City	75 (75%)	85 (85.85%)	0.054
	Village	25 (25%)	14 (14.14%)	
Unpasteurized dairy consumption		3 (3%)	1 (1.01%)	0.62
History of genital infection		4 (4%)	21 (21.20)	0.000
Local dairy consumption history		6 (6%)	7 (7.7%)	0.76
*Listeria monocytogenes*		3 (3%)	0 (0%)	0.08

## Discussion

Infertility and abortion are currently serious problems in countries whose population is declining. It can incur a great financial burden on individuals and society [13, 20, 21]. Infertility in women is defined as not becoming pregnant after a year of having unprotected sex with a fertile male [22], Estimates suggest that 48 million couples and 186 million individuals live with infertility globally [23, 24]. Spontaneous abortion is the termination of pregnancy before the 20^th^-week of pregnancy or the birth of a fetus weighing less than 500 grams [22]. Bacteria such as *Mycoplasma hominis*, *Ureaplasma urealyticum*, *Gardnerella vaginalis*, *L. monocytogenes*, *Neisseria gonorrhoeae,* and *Chlamydia trachomatis* can cause infections or disorders through colonizing in the female reproductive tract [25]. A hypothesis regarding the role of microorganisms in female infertility explains interfering microorganisms in the vagina with sperm function [25]. Infectious agents not only cause infertility by disrupting sperm function but can cause infertility through affecting the different areas of the genital tract [22, 25, 26]. Another hypothesis regarding the role of bacterial infections in spontaneous abortion explains the effect of bacterial phospholipases on increasing the biosynthesis of prostaglandins, indirectly leading to preterm birth and spontaneous abortion [26]. Microbial phospholipases can also hydrolyze phospholipids in the placenta or cell membrane [27, 28]. In a serological study, the prevalence of *L. monocytogenes* was 35.6% in women with spontaneous abortion and 17.5% in women with normal delivery [29]. In another molecular study, the prevalence in women with spontaneous abortion was 14.8% [7]. Another study using culture and molecular methods reported the frequency of infection 7% and 36%, respectively [19]. In 2015, Bahador et al. used molecular methods and reported 14 cases of *L. monocytogenes* among a sample of 170 women with spontaneous abortion [30]. Another study in 2019 reported eight cases of listeriosis in 144 women (5.5%) [31]. In other studies, pregnant women who regularly consumed unpasteurized milk were infected with *L. monocytogenes*, which means pregnant women should avoid foods with a higher risk of contamination with *L*. *monocytogenes* [32]. It is clear that raw milk (unpasteurized) brings greater risk for transmission of bacteria, however, some studies revealed a remarkable prevalence of listeriosis in pregnant women who consumed pasteurized milk [33]. According to the current result, the frequency of infection was 9 (7.7%), of which 4 (3.1%) was observed among women with spontaneous abortion, 2 (1.6%) among women with natural childbirth, and 3 (3%) among fertile women. In a comparison with other studies, the infection was diagnosed using vaginal specimens [34, 35]. According to the current results, the prevalence of the infection in the female population of our study was lower than reported in similar studies, indicating that our study population might have observed preventive measures and enjoyed a higher level of awareness. Furthermore, it is quite noticeable that the applied test in the current study might also have had lower sensitivity and inferior detection limits compared to the ones used in other studies, hence the lower prevalence [31]. Although a few participants had consumed unpasteurized dairy products, it appears that they did not consume raw dairy products and boiled them before consumption. Therefore, a significant reduction in the incidence of listeriosis reported by health centers and obstetricians would be available through increasing the awareness of people including pregnant women regarding to not consume unpasteurized dairy products. This study designed a direct PCR method to detect *L. monocytogenes hlyA* gene on the women vaginal swab specimens; however, it is recommended to detect subtypes of bacteria by using culture and biochemical tests. According to our results, direct PCR is recommended as a primary short-cut method in detection of *L. monocytogenes* in vaginal swab samples. However, regarding the variety of *hlyA* gene sequence, it should be recommended using several genes spontaneously to detect all strains of *L. monocytogenes*.

## Conclusion

The current study revealed no significant relationship between *L. monocytogenes* infection and pregnancy outcomes including spontaneous abortion and infertility. Also, it is recommended to increase the awareness of people, including pregnant women, about not consuming non-pasteurized dairy products, in order to significantly reduce listeriosis and its transmissible methods by health and treatment centers and gynecologists.

## Data Availability

All data generated or analyzed during this study were included in this article but the raw data are available from the corresponding author on reasonable request.

## Abbreviations

PCR: Polymerase Chain Reaction
*L. monocytogenes*: *Listeria monocytogenes*
STIs: Sexually Transmitted Infections
